# Multiomics Profiling and Clustering of Low-Grade Gliomas Based on the Integrated Stress Status

**DOI:** 10.1155/2021/5554436

**Published:** 2021-07-28

**Authors:** Xiaolin Ren, Xin Chen, Chen Zhu, Anhua Wu

**Affiliations:** ^1^Department of Neurosurgery, The First Affiliated Hospital of China Medical University, Shenyang, Liaoning, China; ^2^Department of Neurosurgery, Shenyang Red Cross Hospital, Shenyang, Liaoning, China

## Abstract

**Background:**

Although the prognosis of low-grade glioma is better than that of glioblastoma, there are still some groups with poor prognosis. The integrated stress response contributes to the malignant progress of tumors. As there had limited research focused on the integrated stress status in LGG, it is urgent to profile and reclassify LGG based on the integrated stress response.

**Methods:**

Information of glioma patients was obtained from the Chinese Glioma Genome Atlas, The Cancer Genome Atlas, and the GSE16011 cohorts. Statistical analyses were conducted using GraphPad Prism 8 and R language.

**Results:**

We summarized and quantified four types of integrated stress responses. Relationships between these four types of stress states and the clinical characteristics were analyzed in low-grade glioma. We then reclassified the patients based on these four scores and found that cluster 2 had the worst prognosis, while cluster 1 had the best prognosis. We also established an accurate integrated stress response risk signature for predicting cluster 2. We found that immune response and suppressive immune cell components were more enriched in the high-risk group. We also profiled the genomic differences between the low- and high-risk groups, including the nonmissense mutation of driver genes and the copy number variations.

**Conclusion:**

Low-grade glioma patients were divided into three clusters based on the integrated stress status, with cluster 2 exhibiting malignant transformation trends. The signature adequately reflected the traits of cluster 2, while a high risk score indicated a worse prognosis and an enriched inhibitory immune microenvironment.

## 1. Introduction

Glioma is the most common and deadly primary malignant tumor in the central nervous system [1]. Gliomas can be divided into high-grade glioma and low-grade glioma (LGG). We defined grade 4 glioma as glioblastoma(GBM)[2] and grade 2 and 3 glioma as LGG. GBM is highly aggressive, and even with standardized treatment, the median survival time of patients is only 14.6 months [3]. Compared with GBM, LGG is less aggressive and their progression is slightly slower [4, 5]. However, increasing studies have reported that many LGG patients have a tendency toward malignant transformation, which can transform into higher grades, leading to adverse outcomes [6]. The outcomes of glioma patients are highly variable and heterogeneous [7], even in patients with the same tumor grade. Although isocitrate dehydrogenase 1 (IDH1) mutations and chromosome 1p/19q codeletions have been identified as good prognostic markers for LGG, there is still an urgent need to develop a new classification system for LGG, which can identify LGG patients with a tendency toward malignant transformations.

Studies have shown that the integrated stress response (ISR) is related to the mechanisms and progressions of many complex diseases [8], including cancer, diabetes, and metabolic diseases. The ISR state refers to an evolutionarily conserved intracellular signal network, which helps cells, tissues, and organisms adapt to various stimuli from the microenvironment. The ISR includes protein homeostasis defects, nutritional deficiencies, viral infections, and oxidative stress. The ISR restores cell homeostasis by reediting gene expressions. Different types of stress responses are represented by four special kinases (PERK, GCN2, PKR, and HRI) [9]. The organs most affected in the ISR are the brain and pancreas [10]. Exosomes induced by endoplasmic reticulum stress promote the immune escape of breast cancer by regulating the expression of PD-L1 in macrophages [11]. The redox state affects T cell activation and subsequent supervision of redox-sensitive immune regulatory transcription factors such as NF-*κ*B, NFAT, and AP-1, which are involved in the pathogenesis of inflammation-related diseases [12]. Multiple studies have indicated that the ISR is tightly embedded in the innate immune response of cells, and all four ISR kinases play a role in immunity and inflammation [13].

Most of the carcinogenic pathways found in human cancers cause various forms of protein synthesis disorders, while the ISR controls protein synthesis and protein stability. Three ISR kinases (PKR, PERK, and GCN2) have been correlated with cancer [14], but there has been minimal research describing the status of the ISR in LGG. There is therefore an urgent need to analyze its distribution status and clinical value in LGG, to reclassify LGG from the perspective of integrated stress and to identify subgroups of LGG patients with malignant tendencies.

## 2. Materials and Methods

### 2.1. Data Collection

This study included 1211 LGG patients. Patient information was summarized from four cohorts: The Cancer Genome Atlas (TCGA) RNA-seq cohort (http://www.tcga.org/), the Chinese Glioma Genome Atlas (CGGA) microarray cohort (http://www.cgga.org.cn/), the CGGA RNA sequencing (RNA-seq) cohort (http://www.cgga.org.cn/), and the GSE16011 (http://www.cgga.org.cn/) and the GSE16011 microarray cohort (https://www.ncbi.nlm.nih.gov/geo/query/acc.cgi?acc=GSE16011). The LGG mutation data (MAF file) were downloaded from (https://portal.gdc.cancer.gov/), and copy number variation (CNV) information was acquired from Firehose (http://gdac.broadinstitute.org/).

### 2.2. Summary of Gene Sets

Four gene sets were summarized to represent corresponding biological processes of the ISR. They were endoplasmic reticulum stress (unfolded protein response), viral infection, nutritional deficiency, and oxidative stress. The specific genes of each gene set are shown in Supplementary Table 1.

### 2.3. Classification of LGG Based on the Four Types of ISRs

The four types of integrated stress states were quantified by the ssGSEA algorithm [15], and unsupervised clustering was performed to obtain the most reasonable classification according to these four factors. Survival curves of four clusters of samples were plotted using Prism 8 software (GraphPad, San Diego, CA, USA). Differential genes were screened out using the limma package between the best and the worst clusters in survival quality.

### 2.4. Development and Validation of a ISR-Related Signature Using LASSO Regression Model

The “glmnet” R package was performed to filter the prognosis-related ISR genes by LASSO Cox regression analysis with a ten-fold cross-validation. After identifying the significant genes and their regression coefficients (*β*), we calculated the risk score of each LGG patient by the formula as follows: risk score = ∑_*i*=1_^*N*^Exp_*i*_∗*β*_*i*_.

### 2.5. Function Enrichment Analyses

GSEA (http://www.broadinstitute.org/gsea/index.jsp) was conducted to determine whether the selected gene sets showed statistical differences between different groups. Gene ontology (GO) [16] and Kyoto Encyclopedia of Genes and Genomes (KEGG) [17] pathway enrichment analyses were used to analyze the enriched biological processes. Enriched ontological terms and pathways with *P* < 0.05 were selected and presented in a bubble map using the R package.

### 2.6. Immune Response and Tumor Microenvironment-Related Analyses

The immune cell genes were downloaded from http://cibersort.stanford.edu/ [18], and the immune cell score of each sample was quantified by ssGSEA. Student's *t-*test was used to identify immune cells with significant differences between different groups. Stromal score, immune score, and glioma purity were calculated to evaluate nontumor cells within the microenvironment [19]. The correlations between ISR score and tumor purity and stromal score and immune score were calculated and exhibited. Principal component analysis (PCA) was used to profile the distribution patterns of different groups on the basis of the immune-related transcriptome expression matrix.

### 2.7. Statistical Analysis

SPSS (SPSS, Chicago, IL, USA), Prism 8 (GraphPad), and R 3.6 (https://www. http://r-project.org/) software were used for statistical analyses. Student's *t-*test was used to identify differences in expressions, and Pearson's correlation was used to calculate correlations. A value of *P* < 0.05 was considered to indicate statistical significance (^∗^*P* < 0.05, ^∗∗^*P* < 0.01, ^∗∗∗^P < 0.001, and ^∗∗∗∗^*P* < 0.0001, as indicated in the figures and legends). Low and high-expression groups were classified according to the median expression. Survival analyses were conducted using Kaplan-Meier plotting, and the log-rank test was used to evaluate the differences between stratified groups.

## 3. Results

### 3.1. General Description of the ISR State in LGG patients

**Figure 1:**
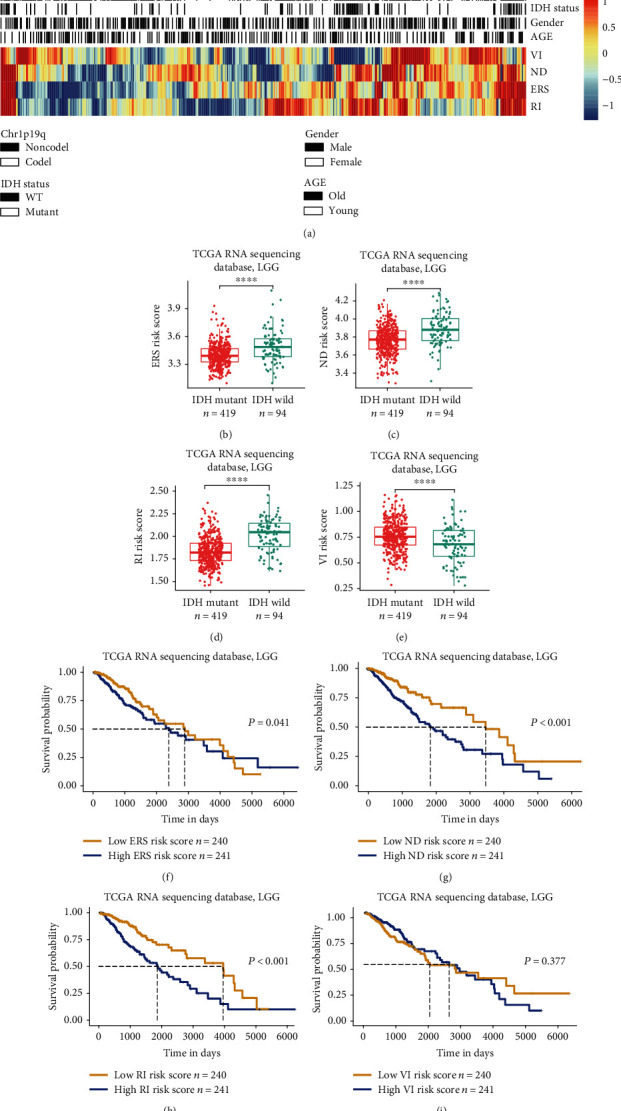
Estimating the clinical value of four integrated stress response- (ISR-) related scores in low-grade glioma (LGG) patients. (a) The expression pattern of the four stress response-related scores with other clinical characteristics of LGG patients. (b–e) High-risk score patients of ERS, ND, and RI were specifically enriched in the IDH1 wild-type gene while low-risk score patients of VI were specifically enriched in the IDH1 wild-type gene in LGGs. (f–h) The high-risk score group of ERS, ND, and RI exhibited an unfavorable prognosis in LGG patients of The Cancer Genome Atlas (TCGA) RNA sequencing cohorts. (i) No prognostic significance of the VI risk score in LGG patients of TCGA RNA sequencing cohorts. ^∗∗∗∗^*P* < 0.0001. ERS: endoplasmic reticulum stress; ND: nutrient deprivation; RI: redox imbalance; VI: viral infection.

The ISR includes the ability to adapt to various stress states in the cell, including the endoplasmic reticulum stress state, nutrient deprivation, viral infection, and redox imbalance. Because few investigators have studied the relationship between ISR and the malignant progression of LGG, we systematically analyzed the clinical value of the ISR in LGGs. We first selected four types of representative gene sets related to the ISR from the official website of GSEA and used the ssGSEA algorithm to quantify the four responses of LGG patients in TCGA, to show the corresponding relationships between the four response scores and LGG clinical characteristics using heat map (Figure 1(a)). We found that endoplasmic reticulum stress, nutritional deprivation, and redox imbalance in the ISR correlated with age, IDH1, 1p19q, and other significant LGG survival prognostic markers. These three stress scores were significantly increased in LGG patients with relatively poor prognoses, but the difference in viral infection scores was not significant. Subsequently, we used the median score as a critical value to analyze the prognostic evaluation value of the four stress states using log-rank survival curves. The results showed that redox state imbalance and the nutritional deprivation state had the most significant impact on the survivals and prognoses of LGG patients, and the survival time of patients in the high scoring group was significantly shortened (Figures 1(f)–1(i)). Patients with the high risk score of the endoplasmic reticulum stress, nutritional deprivation, and redox imbalance were more enriched in the IDH wild (Figures 1(b)–1(d)). Patients with IDH mutations possessed significantly higher viral infection risk score than wild ones (Figure 1(e)).

### 3.2. Reclassification of LGG Patients Based on the Four Types of Stress Response Scores

**Figure 2:**
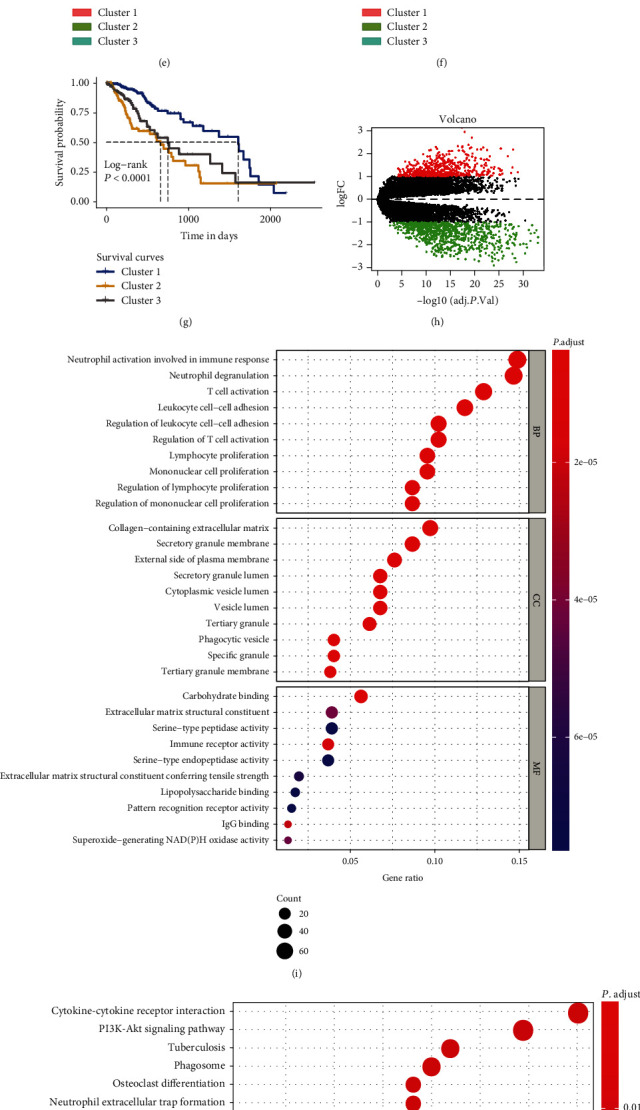
Reclustering and profiling low-grade glioma (LGG) patients based on four integrated stress response- (ISR-) related scores. (a, b) Unsupervised clustering of LGG patients based on four ISR-related scores by using the *k*-means method. (c–f) The distribution patterns of these four ISR-related scores among four clusters. (g) The overall survival analyses among the four cluster samples. (h) Volcano map of the differentially expressed genes between cluster 1 and cluster 2 subtypes. (i) Gene Ontology enrichment of the differentially expressed genes between cluster 1 and cluster 2 subtypes. (j) KEGG enrichment of the differentially expressed genes between the cluster 1 and cluster 2 subtypes.

According to previous results, we performed unsupervised clustering of LGG patients based on the four stress response scores, and the *k*-means clustering results showed that the best distinguishing categories involved three categories (Figures 2(a) and 2(b)). The survival curves of the three types of LGG patients showed that the survival prognosis of cluster 2 was the worst, while the survival prognosis of cluster 1 was the best (Figure 2(g)). We further described different types of ISRs in the three cluster patients using an overlay diagram. Among the cluster 2 subgroups of patients with relatively poor survival prognoses, the endoplasmic reticulum stress state and redox imbalance state scores were relatively high (Figures 2(c) and 2(e)), while the virus infection status score was relatively low. The virus infection stress score was relatively high in the cluster 1 subgroup of patients with relatively good survival prognoses (Figure 2(f)). To further characterize the underlying mechanism of the large differences in survival prognoses between the cluster 1 and cluster 2 subgroups, we screened the differential genes between the two groups of patients (Figure 2(h)) and performed functional enrichment analysis. The enrichment results suggested that cluster 2 was enriched with several immune-related biological processes such as the inflammatory immune response and immune cell chemotaxis (Figures 2(i) and 2(j)). Analysis of the immune microenvironment indicated that inhibitory immune components such as macrophages and neutrophils were more enriched in cluster 2 (Supplementary Fig. 1A).

### 3.3. Establishment of a Reliable ISR Signature That Could Accurately Predict cluster 2 Subtype

**Figure 3:**
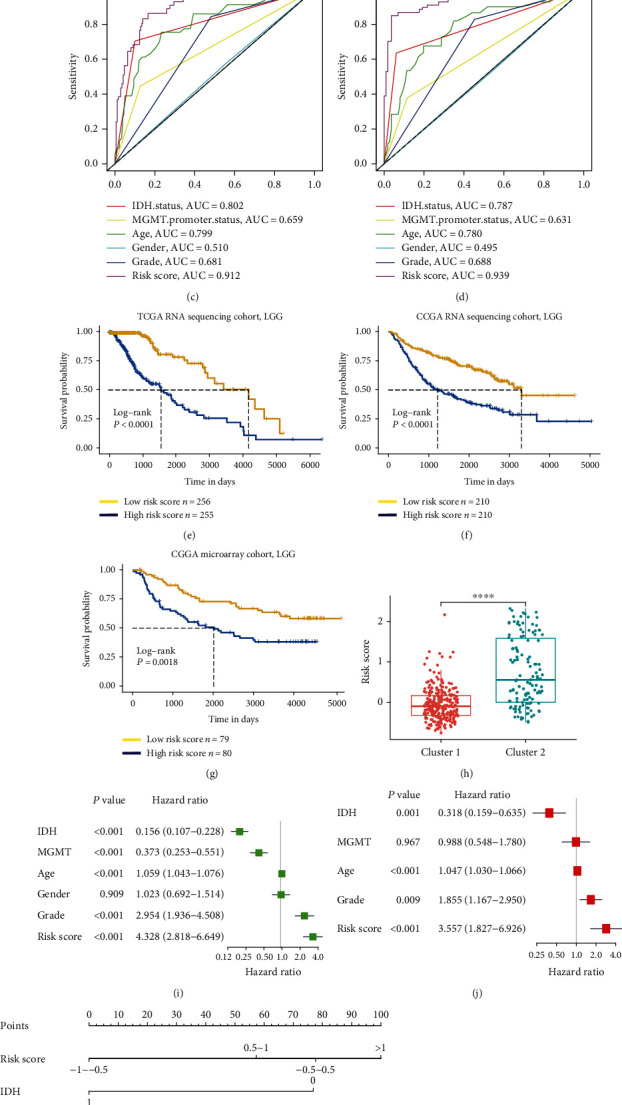
Cluster 2-related integrated stress response (ISR) risk signature is an independent prognostic factor in the low-grade glioma (LGG) cohort. (a) Cross-validation for tuning parameter selection in the proportional hazard model. (b–d) Receiver operating curves of the risk scores to predict 1 year, 2 years, and 3 years of survival in The Cancer Genome Atlas (TCGA) cohort. (e) The high-risk group exhibited strikingly shorter survival times in LGG patients of TCGA RNA sequencing cohorts. (f, g) In the other two validation cohorts, there was also an unfavorable prognosis in the high-risk group. (h) Cluster 1 and cluster 2 exhibited extremely different risk scores. (i, j) Univariate and multivariate Cox analyses of several clinical parameters of the LGG patients in TCGA cohort. (k, l) Nomogram and the calibration plot of the combined model, including the ISR risk signature and other important clinical parameters.

We selected the 127 differentially expressed genes between cluster 1 and cluster 2 with *P* values less than 0.05 and ∣log FC | >2 (Supplementary Table 2). To obtain the best prognostic markers, the LASSO Cox regression analysis was conducted (Figure 3(a)). A total of 11 gene signatures were generated, and the risk scores of the signature based on the regression coefficients were finally calculated (Supplementary Table 4). The ISR risk score of cluster 2 was significantly higher than that of cluster 1 (Figure 3(h)). To estimate the effectiveness of the ISR risk score in predicting prognoses, we constructed a receiver operating characteristic curve (Figures 3(b) – 3(d)) based on 1-year, 2-year, and 3-year survival times and then calculated the area under the curve. The results showed a good predicting efficiency in survival in the discovery cohort. Next, we used the median score as a critical value to analyze the prognostic evaluation value of the three cohorts using the log-rank survival curve. The results showed that among the three cohorts, TCGA-RNA sequencing cohort, CGGA-RNA sequencing cohort, and CGGA-microarray cohort, there were significant differences in survival (*P* < 0.0001). Univariate and multivariate Cox regression analyses were conducted to identify the independent prognostic factors. The results suggested that the ISR risk score could be used as an independent survival-related factor (Figures 3(i) and 3(j)). We obtained the same conclusion in the validation group of the CGGA cohort (Supplementary Table 3). We also established a nomogram to predict the 1-year, 2-year, and 3-year prognoses of LGG patients (Figure 3(k)) and used the calibration chart to evaluate the model (Figure 3(l)). It showed that the ISR risk score was effective in predicting the prognosis of LGG patients.

### 3.4. The High-Risk Group with an Enhanced Local Immune Phenotype

**Figure 4:**
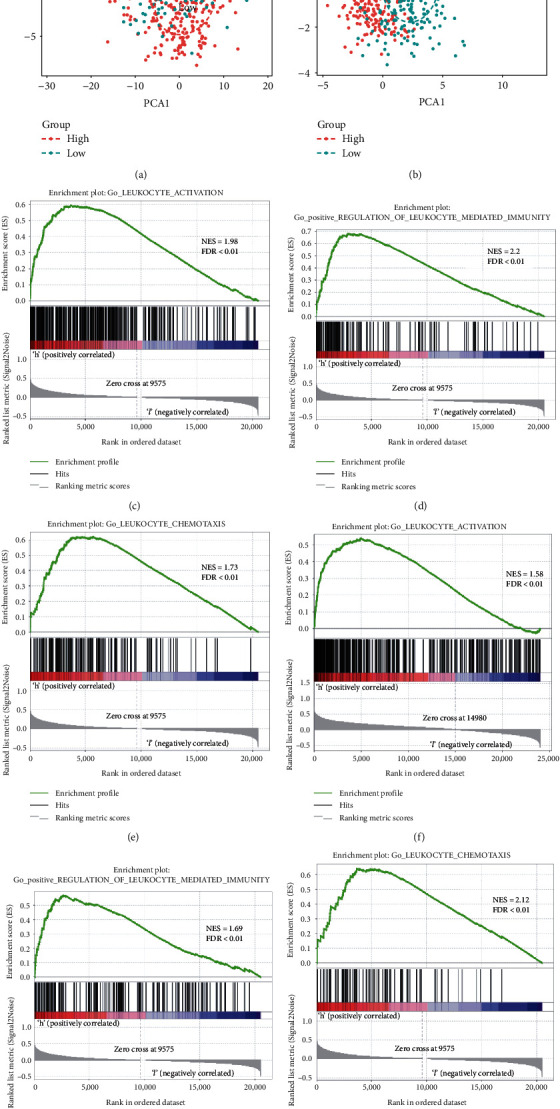
The different immune status of the high- and low-risk groups. (a, b) Principal component analysis showed that the low-grade glioma (LGG) patients in the high- and low-risk groups were distributed in different immune classes. (c–h) GSEA suggested that leukocyte-related processes were enriched in the high-risk group of The Cancer Genome Atlas and Chinese Glioma Genome Atlas RNA sequencing cohorts.

Because cluster 2 was enriched with immune-related biological processes and infiltrated with inhibitory immune cell components, we further characterized the immunophenotypic differences between the high- and low-ISR-risk groups. We first performed PCA based on immune-related genes, which was summarized from the terms of “Immune Response” and “Immune Process” [20, 21]. The results showed that the low- and high-risk group patients were robustly distributed with different locations and patterns, suggesting that low- and high-ISR-risk groups exhibited a totally different immune status (Figures 4(a) and 4(b)). Moreover, we performed GSEA based on a series of immune relevant terms, such as “leukocyte activation,” “leukocyte-mediated immunity,” and “leukocyte chemotaxis.” All these terms were significantly enhanced within the high-risk group (Figures 4(c) – 4(h)). Together, the results showed that the high-risk group was characterized by an enhanced local immune phenotype.

### 3.5. The High-Risk Group Was Infiltrated with an Inhibitory Immune Microenvironment

**Figure 5:**
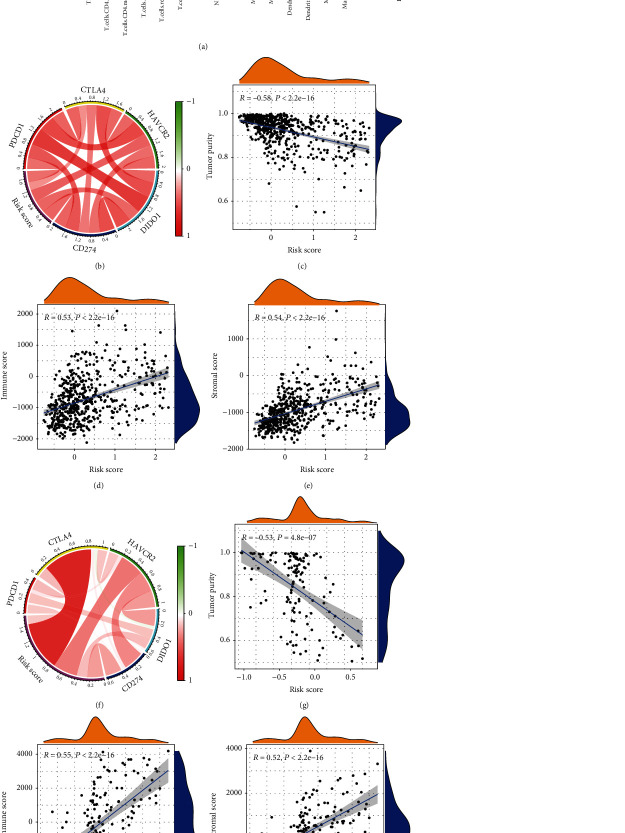
Corresponding relationship between integrated stress response (ISR) risk score and immune microenvironment. (a) The quantified score of the immune gene set showed that patients with a high risk score of integrated stress was highly infiltrated with macrophages of The Cancer Genome Atlas (TCGA) cohort; (b, f) ISR risk score correlated with immunosuppressive checkpoints in the TGGA and Chinese Glioma Genome Atlas (CGGA) sequencing cohorts. (c, g) IRS risk score showed a negative correlation with tumor purity in the TCGA and CGGA RNA sequencing cohorts; (d, e, h, i) The IRS risk score showed a positive correlation with stromal score and immune score in the TCGA and the CGGA RNA sequencing cohorts.

To further characterize the differences of immune microenvironments between the low- and high-risk groups, we downloaded the gene sets data of immune cells from the CIBERSORT website and quantified the immune cell score of each sample using the ssGESA algorithm. The violin diagram shown in Figure 5(a) shows that M0 and M2 type macrophages were more abundant in the high-risk group. In addition, we found that the risk score was positively correlated with the immune score and stromal score, while negatively correlated with the tumor purity (Figures 5(c) and 5(g)). Moreover, the risk score was positively correlated with the expression of several classical inhibitory immune checkpoint-related genes (Figures 5(b) and 5(f)). These results suggested that the high risk score robustly indicated an enhanced local immune response as well as a suppressed immune microenvironment.

### 3.6. Multiomics Differences between Low- and High-ISR-Risk Score Patients by Profiling of Specific Somatic Mutations and Copy Number Variations

**Figure 6:**
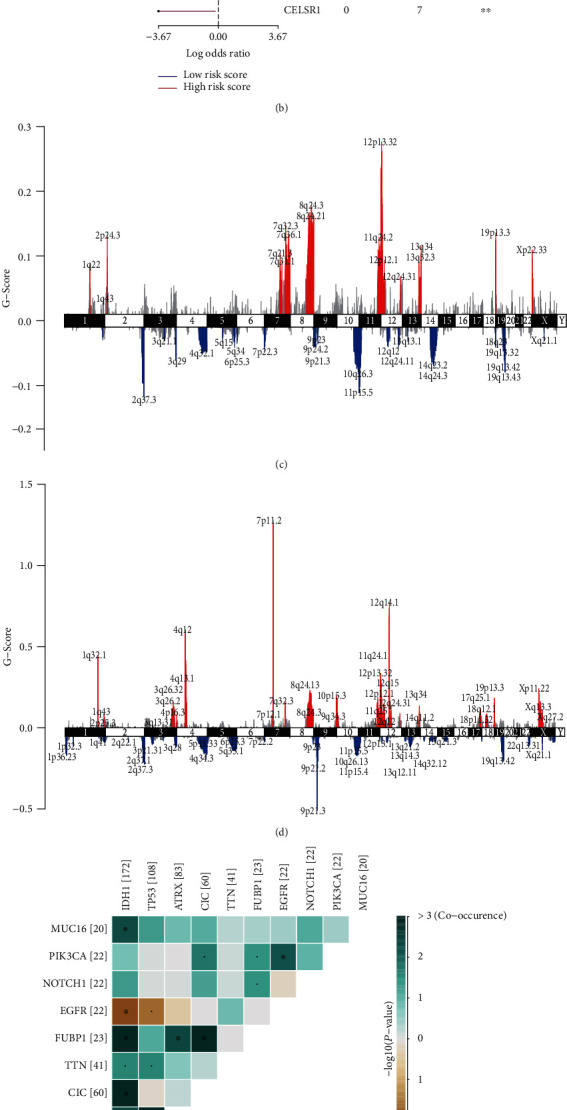
Specific somatic mutations and copy number changes between the high and low integrated stress response (ISR) risk score groups. (a) Low- and high-ISR-risk score group mutation information illustrated in the somatic mutation spectrum. (b) Various mutated genes between the high- and low-IRS-risk score groups. (c, d) Copy number variations based on low- and high-IRS-risk score levels. (e, f) Correlation analyses of mutated genes in the high- and low-risk groups in The Cancer Genome Atlas RNA sequencing cohorts.

To investigate the differences between the high- and low-ISR-risk score groups at the genomic level, we analyzed somatic mutations and copy number variations (CNVs) from the TCGA RNA sequencing cohort. The overall pattern of classical somatic mutations in LGG stratified by the ISR risk score is shown in Figures 6(a) and 6(b)). We found 7 significant somatic mutation genes between the high- and low-risk groups (*P* < 0.05). In the high-risk score group, the somatic mutation profile showed that TTN and CIC had higher mutation frequencies in the high-risk score group.

To identify the specific CNV events of the high- and low-ISR-risk groups, we profiled the copy number variant events between these two groups. The results suggested that the high-risk group had amplification of partial fragments in chromosomes 4, 7, and 12 and partial deletion of fragments in chromosome 9 (Figures 6(c) and 6(d)). Although there was no significant difference in the copy number of important chromosomal fragments such as 1p and 19q between the two groups, amplified genes such as TTN in the high-ISR-risk group may be involved in promoting the malignant progression of LGG.

## 4. Discussion

LGG comprise a group of primary brain tumors caused by glial cells. The LGG patients have a better prognosis than GBM patients ,but they are still incurable, and most of them will relapse or even transform into high-grade gliomas. Previous studies have confirmed that the ISR is related to the mechanisms of a variety of cancers [22, 23]. In addition, there are few studies on the relationship between the ISR and LGG, so there is an urgent need to quantitate and characterize the ISR state in LGG.

By quantitating the four types of stress responses of LGG patients, we found that endoplasmic reticulum stress, nutritional deprivation, and redox imbalance in the ISR were related to age, IDH1, 1p19q, and other LGG significant survival prognostic markers. The above three stress scores were significantly increased in the relatively poor prognoses cluster 2 LGG patients, although the viral infection stress score reflected a different situation. In the majority of human cancers, including breast cancer, pancreatic cancer, lung cancer, skin cancer, prostate cancer, brain cancer, and even liquid malignant tumors, enhanced endoplasmic reticulum stress responses have been found [24]. Studies have shown that continuous activation of the endoplasmic reticulum stress sensor could make malignant cells more tumorigenic, metastatic, and drug-resistant [25, 26]. In addition, the endoplasmic reticulum stress response further hinders the development of protective anti-cancer immunity by regulating the function of myeloid cells in the tumor microenvironment [27]. The endoplasmic reticulum stress state can activate the unfolded protein response, which is necessary for the development of T cells, but the continuous increase of endoplasmic reticulum stress can lead to damages of T cell function, as well as decreased survival [28, 29]. Oxidative stress is the main intracellular signal transducer that maintains autophagy [30]. Once tumors are formed, autophagy allows tumor cells to survive under stress conditions, thereby promoting tumor progression [31, 32]. Regarding nutritional deprivation, there is evidence suggesting that cancer cells can adjust signal pathways to adapt to the new environment and continue to survive [33, 34]. This characteristic of cancer cells is considered to be one of the prerequisites for tumor progression and chemotherapy resistance [35].

Based on these considerations, we know that the occurrence and development of LGG are affected by multiple factors, so a single aspect of stress factors cannot represent its occurrence or development. More angles and more dimensions should be considered in making an overall analysis. We therefore considered grouping the LGG patients in TCGA according to the four kinds of stress factors, with LGG patients divided into three clusters with the best characteristics. Using integrated bioinformatics analyses, we found that in cluster 2, which also exhibited the worst survival condition in these three clusters, the scores of oxidative stress and endoplasmic reticulum stress were significantly higher than those of the other two groups, while the viral infection stress score was significantly lower than those of the other two groups. In cluster 1 with the best prognosis, we found that the viral infection stress score was significantly higher than those of the other two groups, while the oxidative stress risk score was significantly lower than those of the other two groups. Importantly, the nutritional deprivation stress score in the cluster 3 group was significantly lower than that of cluster 1 groups, and the viral stress score was also very high (but with no significant difference compared with cluster 2). However, the prognosis of cluster 3 was significantly worse than that of cluster 1. This showed that redox imbalance stress may play an absolute role in cluster 3, suggesting that the occurrence and development of LGG might be regulated by multiple factors.

The classification that we established based on four kinds of ISR is relatively difficult to implement in specific clinical cases. To make our research results easier for clinical use, we established an integrated stress response-related risk score to represent the traits of cluster 2. Based on this score, the LGG patients in the other two databases were divided into the high- and low-risk groups, resulting in a significant difference (*P* < 0.0001), which was also a good independent factor for survival predictions. Using PCA of TCGA database, we found significant differences in immune functions between the high-risk and low-risk groups. Immunosuppressive cells such as M2, M0, regulatory T cells, and neutrophils were more enriched in the high-ISR-risk score group, which indicated that the reorganization of the immune microenvironment may have contributed to the poor survival of the high-risk group. Similarly, in the validation group of CGGA cohort, we found that immune infiltration and cell chemotaxis were also more enriched in high-risk patients (Supplementary Fig. 3A-B). We also found that the ISR risk score was positively correlated with the expressions of immune checkpoint genes, which further validated our hypothesis that high ISR risk was involved in immune suppression.

We analyzed the gene mutations and copy number variations between the high-risk and low-risk groups and found that the high-risk group had a higher frequency of CIC and TTN mutations. Some report that TTN was at the top ranking of mutated genes in multiple solid tumors, including the gastric adenocarcinoma, small cell lung cancer, and colorectal adenocarcinoma. TTN mutation could also be used to represent increased tumor mutation burden (TMB) and correlated with objective response to immune checkpoint blockade (ICB). [36, 37]. Bettegowda et al.'s study indicated that there was a unique relationship between recurrent chromosomal aberrations and mutations in CIC and suggested that mutations in CIC were key events in the development of oligodendrogliomas [38].

While we profiled copy number variations, we found that the high-risk score group had an amplification of chromosomes 4, 7, and 12. We performed functional analyses of the corresponding gene set and found that it was enriched in angiogenesis and response to external stimuli (Supplementary Fig. 2H).

## 5. Conclusions

Our study provided new insights into the relationships between the integrated stress responses and clinical traits of LGG patients. Furthermore, we identified cluster 1 with the most potential for malignant transformation, which exhibited the worst prognosis and inhibitory immune microenvironment. More importantly, we designed an ISR signature to predict cluster 2, which may facilitate the classification and prediction of individual LGG patients, although the model needs to be further validated in prospective studies and multicenter clinical trials.

## Data Availability

This study included 1211 lower grade glioma (LGG) patients. Patients were summarized from four cohorts: The Cancer Genome Atlas (TCGA) RNA-seq cohort (http://www.tcga.org/), the Chinese Glioma Genome Atlas (CGGA) microarray cohort (http://www.cgga.org.cn/), CGGA RNA sequencing (RNA-seq) cohort (http://www.cgga.org.cn/), and GSE16011 microarray cohort (https://www.ncbi.nlm.nih.gov/geo/query/acc.cgi?acc=GSE16011).

## Abbreviations

LGG: Low-grade glioma CNVs: Copy number variations PCA: Principal component analysis
GBM: Glioblastoma
ISR: Integrated stress response
TCGA: The Cancer Genome Atlas
CGGA: The Chinese Glioma Genome Atlas
GSEA: Gene Set Enrichment Analysis
GEO: Gene expression omnibus.

## Supplementary Materials

Supplementary 1 Table 1: integrated stress response-related genes.

Supplementary 2 Table 2: differential genes between cluster 1 and cluster 2.

Supplementary 3 Table 3: univariate and multivariate analyses of CGGA.

Supplementary 4 Table 4: the 11-gene IRS-related signature.

Supplementary 5 Figure 1: the high- and low-risk groups were associated with different immune states.

Supplementary 6 Figure 2: the predictive accuracy of integrated stress risk model for LGG prognosis.

Supplementary 7 Figure 3: GO and KEGG enrichment (CGGA).

## References

[1] Ostrom Q. T., Gittleman H., Liao P. (2017). CBTRUS statistical report: primary brain and other central nervous system tumors diagnosed in the United States in 2010-2014. *Neuro-Oncology*.

[2] Nizamutdinov D., Stock E. M., Dandashi J. A. (2018). Prognostication of survival outcomes in patients diagnosed with glioblastoma. *World Neurosurgery*.

[3] Khasraw M., Reardon D. A., Weller M., Sampson J. H. (2020). PD-1 inhibitors: do they have a future in the treatment of glioblastoma?. *Clinical Cancer Research*.

[4] Zhang X., Lu X., Liu Z. (2019). Integrating multiple-level molecular data to infer the distinctions between glioblastoma and lower-grade glioma. *International Journal of Cancer*.

[5] Chammas M., Saadeh F., Maaliki M., Assi H. (2019). Therapeutic interventions in adult low-grade gliomas. *Journal of Clinical Neurology*.

[6] Duffau H., Taillandier L. (2015). New concepts in the management of diffuse low-grade glioma: proposal of a multistage and individualized therapeutic approach. *Neuro-Oncology*.

[7] Liu Z., Zhang T., Jiang H., Xu W., Zhang J. (2019). Conventional MR-based preoperative nomograms for prediction of IDH/1p19q subtype in low-grade glioma. *Academic Radiology*.

[8] Costa-Mattioli M., Walter P. (2020). The integrated stress response: from mechanism to disease. *Science*.

[9] Wek R. C. (2018). Role of eIF2*α* kinases in translational control and adaptation to cellular stress. *Cold Spring Harbor Perspectives in Biology*.

[10] Abdulkarim B., Nicolino M., Igoillo-Esteve M. (2015). A missense mutation in PPP1R15B causes a syndrome including diabetes, short stature, and microcephaly. *Diabetes*.

[11] Yao X., Tu Y., Xu Y., Guo Y., Yao F., Zhang X. (2020). Endoplasmic reticulum stress-induced exosomal miR‐27a‐3p promotes immune escape in breast cancer via regulating PD-L1 expression in macrophages. *Journal of Cellular and Molecular Medicine*.

[12] Gambhir L., Sharma V., Kandwal P., Saxena S. (2019). Perturbation in cellular redox homeostasis: decisive regulator of T cell mediated immune responses. *International Immunopharmacology*.

[13] Cláudio N., Dalet A., Gatti E., Pierre P. (2013). Mapping the crossroads of immune activation and cellular stress response pathways. *The EMBO Journal*.

[14] Pavon-Eternod M., Gomes S., Rosner M. R., Pan T. (2013). Overexpression of initiator methionine tRNA leads to global reprogramming of tRNA expression and increased proliferation in human epithelial cells. *RNA*.

[15] Barbie D. A., Tamayo P., Boehm J. S. (2009). Systematic RNA interference reveals that oncogenic KRAS-driven cancers require TBK1. *Nature*.

[16] The Gene Ontology Consortium (2017). Expansion of the Gene Ontology knowledgebase and resources. *Nucleic Acids Research*.

[17] Du J., Yuan Z., Ma Z., Song J., Xie X., Chen Y. (2014). KEGG-PATH: Kyoto encyclopedia of genes and genomes-based pathway analysis using a path analysis model. *Molecular BioSystems*.

[18] Li B., Severson E., Pignon J. C. (2016). Comprehensive analyses of tumor immunity: implications for cancer immunotherapy. *Genome Biology*.

[19] Zhang C., Cheng W., Ren X. (2017). Tumor purity as an underlying key factor in glioma. *Clinical Cancer Research*.

[20] Loyal L., Warth S., Jürchott K. (2020). SLAMF7 and IL-6R define distinct cytotoxic versus helper memory CD8(+) T cells. *Nature Communications*.

[21] O’Melia M. J., Rohner N. A., Manspeaker M. P., Francis D. M., Kissick H. T., Thomas S. N. (2020). Quality of CD8(+) T cell immunity evoked in lymph nodes is compartmentalized by route of antigen transport and functional in tumor context. *Science Advances*.

[22] Dey S., Sayers C. M., Verginadis I. I. (2015). ATF4-dependent induction of heme oxygenase 1 prevents anoikis and promotes metastasis. *The Journal of Clinical Investigation*.

[23] Lin W., Lin Y., Li J., Harding H. P., Ron D., Jamison S. (2011). A deregulated integrated stress response promotes interferon-*γ*-induced medulloblastoma. *Journal of Neuroscience Research*.

[24] Han J., Kaufman R. J. (2017). Physiological/pathological ramifications of transcription factors in the unfolded protein response. *Genes & Development*.

[25] Bahar E., Kim J. Y., Yoon H. (2019). Chemotherapy resistance explained through endoplasmic reticulum stress-dependent signaling. *Cancers*.

[26] Gan P. P., Zhou Y. Y., Zhong M. Z., Peng Y., Li L., Li J. H. (2018). Endoplasmic reticulum stress promotes autophagy and apoptosis and reduces chemotherapy resistance in mutant p 53 lung cancer cells. *Cellular Physiology and Biochemistry*.

[27] Cubillos-Ruiz J. R., Bettigole S. E., Glimcher L. H. (2017). Tumorigenic and immunosuppressive effects of endoplasmic reticulum stress in cancer. *Cell*.

[28] Solanki N. R., Stadanlick J. E., Zhang Y. (2016). Rpl 22 loss selectively impairs *αβ* T cell development by dysregulating endoplasmic reticulum stress signaling. *Journal of Immunology*.

[29] Kamimura D., Katsunuma K., Arima Y. (2015). KDEL receptor 1 regulates T-cell homeostasis via PP1 that is a key phosphatase for ISR. *Nature Communications*.

[30] Filomeni G., De Zio D., Cecconi F. (2015). Oxidative stress and autophagy: the clash between damage and metabolic needs. *Cell Death and Differentiation*.

[31] Li J., Chen X., Kang R., Zeh H., Klionsky D. J., Tang D. (2020). Regulation and function of autophagy in pancreatic cancer. *Autophagy*.

[32] Das C. K., Banerjee I., Mandal M. (2020). Pro-survival autophagy: an emerging candidate of tumor progression through maintaining hallmarks of cancer. *Seminars in Cancer Biology*.

[33] Mondal S., Bhattacharya K., Mandal C. (2018). Nutritional stress reprograms dedifferention in glioblastoma multiforme driven by PTEN/Wnt/Hedgehog axis: a stochastic model of cancer stem cells. *Cell Death Discovery*.

[34] Morris S. L., Huang S. (2016). Crosstalk of the Wnt/*β*-catenin pathway with other pathways in cancer cells. *Genes & Diseases*.

[35] Ahmadiankia N. (2020). In vitro and in vivo studies of cancer cell behavior under nutrient deprivation. *Cell Biology International*.

[36] DePristo M. A., Banks E., Poplin R. (2011). A framework for variation discovery and genotyping using next-generation DNA sequencing data. *Nature Genetics*.

[37] Jia Q., Wang J., He N., He J., Zhu B. (2019). Titin mutation associated with responsiveness to checkpoint blockades in solid tumors. *JCI Insight*.

[38] Bettegowda C., Agrawal N., Jiao Y. (2011). Mutations in CIC and FUBP1 contribute to human oligodendroglioma. *Science*.

