# Supplementary material for: Multiomics Profiling and Clustering of Low-Grade Gliomas Based on the Integrated Stress Status
Source: Biomed Res Int. 2021 Jul 28;2021:5554436. doi: 10.1155/2021/5554436 (PMC8343268; doi:10.1155/2021/5554436)
Supplement: Supplementary 1 — Table 1: integrated stress response-related genes. [file 5554436.f1.docx]

Supplementary table 1 Integrated stress response-related genes

| Endoplasmic reticulum-related stress genes | Viral infection-reatled genes | Nutrient deprivation-related genes | Redox homeostasis-related gene |
| --- | --- | --- | --- |
| ACADVL | ABCA4 | AKR1C3 | APEX1 |
| ADD1 | ABL1 | ALB | CYBA |
| AGR2 | ACAA1 | AMBRA1 | CYBB |
| AMFR | ACAT2 | AOC1 | DDIT3 |
| ARFGAP1 | ACBD4 | ASNS | DLD |
| ASNS | ADAM33 | ATF3 | DNAJC10 |
| ATF3 | ADAMTS6 | ATF4 | DNAJC16 |
| ATF4 | AGFG2 | ATG14 | EGLN2 |
| ATF6 | AGTR1 | ATG5 | ERO1A |
| ATF6B | AHSP | ATG7 | ERO1B |
| ATP6V0D1 | ANGPTL2 | BCL2 | ERP44 |
| BAK1 | APCDD1 | BECN1 | GCLC |
| BAX | APH1B | BECN2 | GLRX |
| BCL2L11 | AR | BHLHA15 | GLRX2 |
| BFAR | ARVCF | BMPR2 | GLRX3 |
| BHLHA15 | ATP8B2 | BMT2 | GLRX5 |
| BOK | BANK1 | C12orf66 | GPX1 |
| CALR | BMF | CADPS2 | GRXCR1 |
| CALR3 | BMP4 | CARTPT | GSR |
| CANX | BTBD2 | CDKN1A | HVCN1 |
| CCL2 | BTNL9 | CLEC16A | KRIT1 |
| CCND1 | C11orf95 | COMT | LPO |
| CDK5RAP3 | C12orf49 | CPEB4 | MPO |
| CLGN | C16orf70 | CTSV | NCF1 |
| COPS5 | C17orf75 | DAP | NCF2 |
| CREB3 | C1orf100 | DAPL1 | NCF4 |
| CREB3L1 | C4orf47 | DEPDC5 | NFE2L2 |
| CREB3L2 | CAMKK1 | DNAJC15 | NHLRC2 |
| CREB3L3 | CANT1 | DSC2 | NME8 |
| CREB3L4 | CBX2 | EHMT2 | NME9 |
| CREBRF | CCDC61 | EIF2AK2 | NNT |
| CTDSP2 | CD248 | EIF2AK3 | NOS1 |
| CTH | CD300LG | EIF2AK4 | NOS2 |
| CUL7 | CD5L | EIF2S1 | NOS3 |
| CXCL8 | CENPH | FADS1 | NQO1 |
| CXXC1 | CENPP | FAS | NXN |
| DAB2IP | CHDH | FBXO22 | NXNL1 |
| DCTN1 | CHIC1 | FNIP1 | P4HB |
| DDIT3 | CHRDL2 | FOXA3 | PDIA2 |
| DDX11 | CHST12 | FOXO1 | PDIA3 |
| DERL1 | CLDN22 | GABARAP | PDIA4 |
| DERL2 | CNIH2 | GABARAPL1 | PDIA5 |
| DERL3 | CNTLN | GABARAPL2 | PDIA6 |
| DNAJB11 | COMMD9 | GABARAPL3 | PDILT |
| DNAJB9 | COX6B2 | GAS2L1 | PRDX1 |
| DNAJC3 | CPT1C | GAS6 | PRDX2 |
| EDEM1 | CTIF | GBA | PRDX3 |
| EIF2AK2 | CUBN | GCN1 | PRDX4 |
| EIF2AK3 | CYB561A3 | GLUL | PRDX5 |
| EIF2S1 | CYP27A1 | HFE | PRDX6 |
| EP300 | CYP2E1 | HIGD1A | PTGES2 |
| ERN1 | CYP2F1 | HNRNPA1 | QSOX1 |
| ERO1A | DDAH2 | HNRNPL | QSOX2 |
| EXTL1 | DHODH | HSPA5 | RAC2 |
| EXTL2 | DLG2 | HSPA8 | SCO2 |
| EXTL3 | DLK2 | IFI16 | SELENOS |
| FGF21 | DYNC2LI1 | IMPACT | SELENOT |
| FICD | DZIP1L | INHBB | SH3BGRL3 |
| FKBP14 | ECHDC3 | ITFG2 | SLC11A1 |
| GET3 | EDEM2 | JUN | TMX1 |
| GFPT1 | EGLN2 | KIAA1324 | TMX2 |
| GOSR2 | EMCN | KLF10 | TMX3 |
| GSK3A | FABP4 | KPTN | TMX4 |
| HDGF | FAM83D | KRT20 | TXN |
| HERPUD1 | FCSK | LAMP2 | TXN2 |
| HERPUD2 | FGD5 | LARS1 | TXNDC11 |
| HSP90B1 | FGFR2 | LRRK2 | TXNDC12 |
| HSPA5 | FKBP1B | MAP1LC3A | TXNDC15 |
| HYOU1 | FMO5 | MAP1LC3B | TXNDC16 |
| IGFBP1 | FNDC10 | MAP1LC3B2 | TXNDC2 |
| KDELR3 | FZD2 | MAP1LC3C | TXNDC5 |
| KLHDC3 | GANC | MAP3K5 | TXNDC8 |
| LMNA | GJA4 | MAPK1 | TXNDC9 |
| MBTPS1 | GLIS2 | MAPK3 | TXNL1 |
| MBTPS2 | GNPDA1 | MAPK8 | TXNRD1 |
| MYDGF | GREB1L | MARS1 | TXNRD2 |
| NCK1 | GRIK5 | MAX | TXNRD3 |
| NCK2 | GRK4 | MIOS |  |
| NFE2L2 | GSTT1 | MTMR3 |  |
| PARP16 | GTF3C1 | MTOR |  |
| PARP6 | GUCY1B1 | MYBBP1A |  |
| PARP8 | HCAR1 | MYH13 |  |
| PDIA5 | HOXB3 | MYOD1 |  |
| PDIA6 | HOXD9 | NFE2L2 |  |
| PIK3R1 | HS1BP3 | NPRL2 |  |
| PLA2G4B | HSCB | NPRL3 |  |
| PPP1R15A | IFT140 | NUAK2 |  |
| PPP1R15B | IFT46 | NUPR2 |  |
| PPP2R5B | IL20RB | PCK1 |  |
| PREB | INHA | PCSK9 |  |
| PTPN1 | INTS9 | PDK4 |  |
| PTPN2 | ISLR2 | PICK1 |  |
| RACK1 | KCNC1 | PIK3C2B |  |
| RHBDD2 | KLF15 | PIK3C3 |  |
| SEC31A | LAMA1 | PIK3R4 |  |
| SELENOS | LASP1 | PMAIP1 |  |
| SERP1 | LENG8 | PPM1D |  |
| SERP2 | LGI4 | PRKAA1 |  |
| SHC1 | LHFPL4 | PRKAA2 |  |
| SRPRA | LOXL3 | PRKD1 |  |
| SRPRB | LRRC17 | RALB |  |
| SSR1 | LTC4S | RIPOR1 |  |
| STC2 | LTO1 | RNF152 |  |
| STUB1 | LYPLAL1 | RPTOR |  |
| SULT1A3 | MAP6 | RRAGA |  |
| SULT1A4 | MARVELD1 | RRAGB |  |
| SYVN1 | MAVS | RRAGC |  |
| TATDN2 | MERTK | RRAGD |  |
| TBL2 | MESP2 | RRP8 |  |
| TLN1 | MFSD11 | SEH1L |  |
| TMEM33 | MFSD12 | SESN1 |  |
| TPP1 | MITF | SESN2 |  |
| TSPYL2 | MMP11 | SESN3 |  |
| VAPB | MMS19 | SFRP1 |  |
| VCP | MRGPRF | SH3GLB1 |  |
| WFS1 | MRNIP | SIRT1 |  |
| WIPI1 | MYO7A | SLC2A1 |  |
| XBP1 | NDE1 | SLC38A2 |  |
| YIF1A | NICN1 | SLC38A3 |  |
| YOD1 | NOVA1 | SLC39A4 |  |
| ZBTB17 | NPEPL1 | SLC39A5 |  |
|  | NPR2 | SRD5A1 |  |
|  | NT5C2 | SREBF1 |  |
|  | OLFML1 | SREBF2 |  |
|  | OPHN1 | STK24 |  |
|  | OSR2 | STK26 |  |
|  | OXTR | SZT2 |  |
|  | PABPC4L | TBL2 |  |
|  | PABPC5 | TNRC6A |  |
|  | PAQR4 | TP53 |  |
|  | PBLD | UPP1 |  |
|  | PCSK7 | USP33 |  |
|  | PEPD | WDR24 |  |
|  | PLEKHA7 | WDR45 |  |
|  | PLEKHG5 | WDR45B |  |
|  | PLEKHH3 | WDR59 |  |
|  | POLI | WIPI1 |  |
|  | POU4F1 | WIPI2 |  |
|  | PROCA1 | WNT2B |  |
|  | PRR12 | WNT4 |  |
|  | PRRG3 | WNT9B |  |
|  | PXMP4 | WRN |  |
|  | PYGB | XBP1 |  |
|  | RANBP6 | XPR1 |  |
|  | RASGRF2 | ZC3H12A |  |
|  | RDM1 | ZFYVE1 |  |
|  | REXO5 |  |  |
|  | RHEBL1 |  |  |
|  | RUNX1T1 |  |  |
|  | SESN1 |  |  |
|  | SFXN3 |  |  |
|  | SIRT2 |  |  |
|  | SKA2 |  |  |
|  | SKI |  |  |
|  | SLC25A23 |  |  |
|  | SLC27A1 |  |  |
|  | SLC29A3 |  |  |
|  | SLC38A9 |  |  |
|  | SLC45A3 |  |  |
|  | SLC46A3 |  |  |
|  | SNCAIP |  |  |
|  | SNN |  |  |
|  | SNX21 |  |  |
|  | SNX33 |  |  |
|  | SPICE1 |  |  |
|  | SPOCK2 |  |  |
|  | SRGAP3 |  |  |
|  | SRPX2 |  |  |
|  | STC2 |  |  |
|  | SYT17 |  |  |
|  | TBL2 |  |  |
|  | TCAF1 |  |  |
|  | TCF19 |  |  |
|  | TFAP2E |  |  |
|  | TM7SF2 |  |  |
|  | TNFRSF25 |  |  |
|  | TNRC6C |  |  |
|  | TNS2 |  |  |
|  | TP73 |  |  |
|  | TRIM37 |  |  |
|  | TRMU |  |  |
|  | TRPC3 |  |  |
|  | TTC12 |  |  |
|  | TTC21B |  |  |
|  | TTC38 |  |  |
|  | TUSC2 |  |  |
|  | TYK2 |  |  |
|  | UCN2 |  |  |
|  | USP54 |  |  |
|  | VHL |  |  |
|  | VPS33B |  |  |
|  | VSIG4 |  |  |
|  | WDR13 |  |  |
|  | WDR90 |  |  |
|  | YPEL4 |  |  |
|  | ZBTB8A |  |  |
|  | ZFP30 |  |  |
|  | ZFYVE19 |  |  |
|  | ZHX3 |  |  |
|  | ZNF862 |  |  |
