# Supplementary material for: Multiomics Profiling and Clustering of Low-Grade Gliomas Based on the Integrated Stress Status
Source: Biomed Res Int. 2021 Jul 28;2021:5554436. doi: 10.1155/2021/5554436 (PMC8343268; doi:10.1155/2021/5554436)
Supplement: Supplementary 2 — Table 2: differential genes between cluster 1 and cluster 2. [file 5554436.f2.docx]

**Supplementary table 2 :Differential genes between cluster1 and cluster2(|LogFC|≧2)**

*Highlined in red are the sets of genes identified by Lasso.

| SFRP2 | CDK5R2 | GRM4 | LRTM2 | SNAP25 |
| --- | --- | --- | --- | --- |
| KCNK3 | CELF4 | HAR1A | LTF | SNAP91 |
| ABCC3 | CHGA | HMP19 | MAL2 | SNCB |
| HOXA7 | CHI3L2 | HOXA10 | MPPED1 | SOHLH1 |
| COL8A1 | CHRM1 | HOXA4 | MYT1L | SPHKAP |
| SERPINA5 | CKMT1A | HOXA5 | NEFL | SPOCD1 |
| HOXA1 | CLEC5A | HPD | NEFM | SRPX2 |
| CHI3L1 | CP | HRH3 | NELL1 | SRRM4 |
| METTL7B | CPLX2 | HRNBP3 | NEUROD2 | SSTR1 |
| IRX5 | CSMD3 | HTR1A | NEUROD6 | STYK1 |
| SHOX2 | CTXN3 | HTR5A | NGB | SULT4A1 |
| ACTL6B | CUX2 | IGFN1 | NRGN | SVOP |
| ANXA1 | DACH2 | INA | OSR2 | SYCE1 |
| ATP2B3 | DDIT4L | INSM2 | PACSIN1 | SYN2 |
| ATP8A2 | DDN | IQSEC3 | PCSK2 | SYT13 |
| C19orf30 | EGR4 | JPH3 | PDPN | SYT4 |
| C1QL3 | EIF4E1B | KCNB2 | PRKCG | TLX1 |
| C2orf85 | FAM123C | KCNC2 | PRLHR | TMEM132D |
| C7orf57 | FAM163B | KCNT1 | PVALB | TMEM155 |
| CABP1 | FAM19A1 | KCNV1 | RIMS2 | TNFSF12.TNFSF13 |
| CACNG2 | GABRA1 | KIAA0748 | SCRT1 | TNNT2 |
| CACNG3 | GABRA4 | KIAA1239 | SCRT2 | TRIM67 |
| CALY | GABRB2 | KIAA1486 | SLC12A5 | UNC13C |
| CAMK2A | GABRG2 | KIAA1644 | SLC17A6 | VSNL1 |
| CBLN1 | GLP1R | KLK7 | SLC17A7 | VSTM2A |
| CBLN2 | GLRA3 | KLRC2 | SLC1A6 | WDR38 |
| CCK | GPR26 | L1CAM | SLC30A3 | WNT7B |
| CCKBR | GPR6 | LHX5 | SLC47A2 | WSCD2 |
| CDH18 | GRIN1 | LOC157627 | SLC6A7 | XKR7 |
| CDH9 | GRIN3A | LPPR3 | SLC8A2 |  |
