# Supplementary material for: Multiomics Profiling and Clustering of Low-Grade Gliomas Based on the Integrated Stress Status
Source: Biomed Res Int. 2021 Jul 28;2021:5554436. doi: 10.1155/2021/5554436 (PMC8343268; doi:10.1155/2021/5554436)
Supplement: Supplementary 3 — Table 3: univariate and multivariate analyses of CGGA. [file 5554436.f3.docx]

| Supplementary table 3 Univariate and multivariate analyses of recurrence-free survival in low grade glioma patients(CGGA RNA sequencing cohorts)  **Univariate analysis** **Multivariate analysis** | | | | |
| --- | --- | --- | --- | --- |
| **Variable** | **P value** | **HR** | **P value** | **HR** |
| IDH Status | <0.001 | 0.471(0.346-0.642) | <0.001 | 0.484(0.355-0.661) |
| MGMT promoter Status | 0.178 | 0.807(0.590-1.103) |  |  |
| WHO grade | <0.01 | 2.634(1.908-3.637) | <0.001 | 2.950(2.108-4.129) |
| Gender | 0.687 | 1.060(0.799-1.407) |  |  |
| Riskscore | <0.01 | 3.138(2.128-4.245) | <0.001 | 3.235(2.304-4.675) |

**Notes:** “–” indicates the factors were not included in multivariate analysis due to no significance in univariate analysis.
