# Supplementary material for: Multiomics Profiling and Clustering of Low-Grade Gliomas Based on the Integrated Stress Status
Source: Biomed Res Int. 2021 Jul 28;2021:5554436. doi: 10.1155/2021/5554436 (PMC8343268; doi:10.1155/2021/5554436)
Supplement: Supplementary 4 — Table 4: the 11-gene IRS-related signature. [file 5554436.f4.docx]

| Supplementary Table4 : The 11-gene IRS related signature | | |
| --- | --- | --- |
|  | Symbol | beta |
| 1 | SFRP2 | 0.002 |
| 2 | KCNK3 | 0.0278 |
| 3 | ABCC3 | 0.0105 |
| 4 | HOXA7 | 0.0174 |
| 5 | COL8A1 | 0.0061 |
| 6 | SERPINA5 | 0.0518 |
| 7 | HOXA1 | -0.0253 |
| 8 | CHI3L1 | 0.0514 |
| 9 | METTL7B | 0.0136 |
| 10 | IRX5 | -0.0598 |
| 11 | SHOX2 | 0.0987 |
