# Supplementary material for: Multiomics Profiling and Clustering of Low-Grade Gliomas Based on the Integrated Stress Status
Source: Biomed Res Int. 2021 Jul 28;2021:5554436. doi: 10.1155/2021/5554436 (PMC8343268; doi:10.1155/2021/5554436)
Supplement: Supplementary 5 — Figure 1: the high- and low-risk groups were associated with different immune states. [file 5554436.f5.pdf]

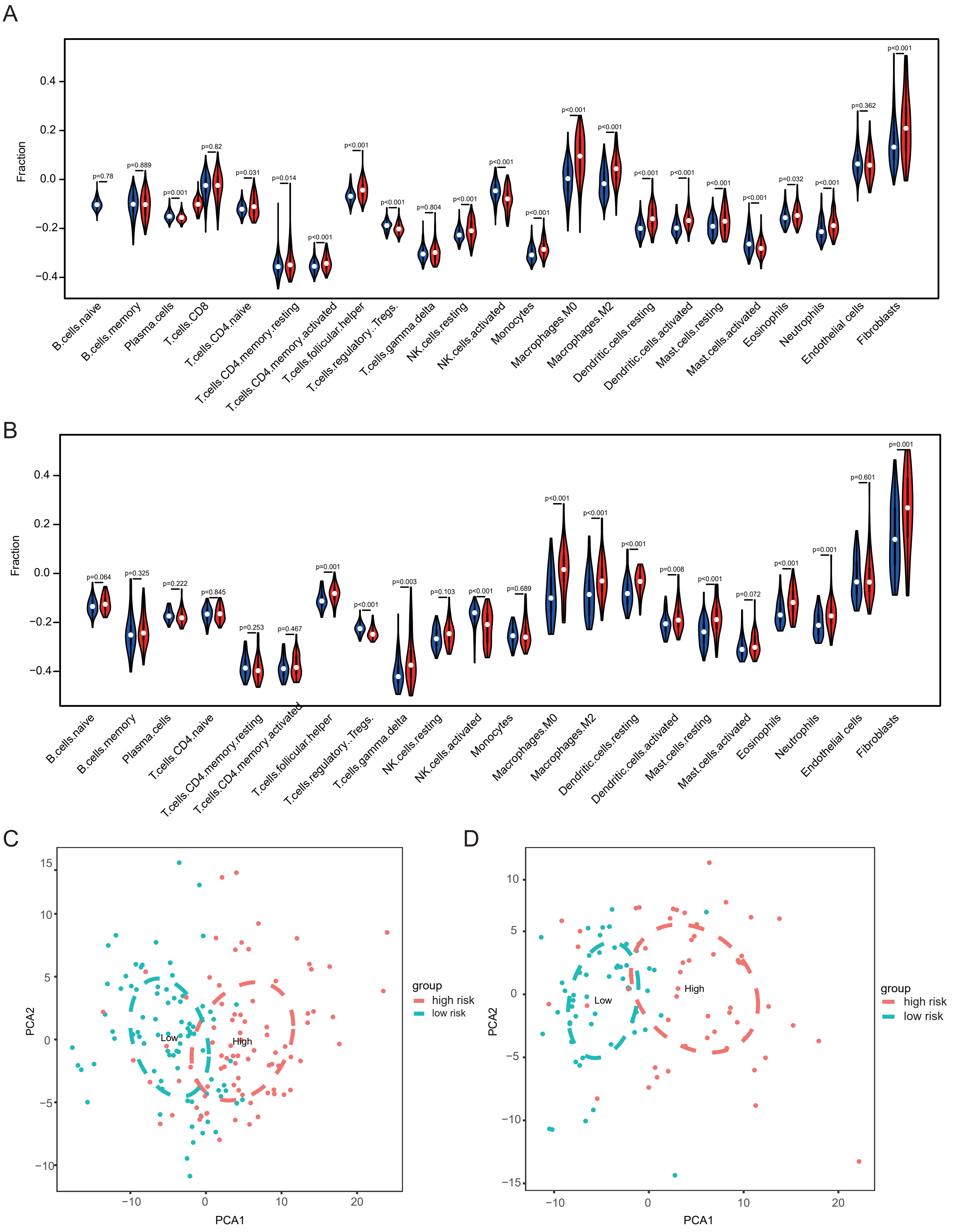

Supplementary Figure 1 The high and low risk groups were associated with different immune states. (A): The quantified score of immune gene set showed that patients with Cluster 2 were more enrichment in macrophages than Cluster 1. (B). The quantified score of immune gene set showed that patients with high risk score of integrated stress was associated with macrophages in CGGA. (C-D) PCA showed that the LGG patients in the high and low-risk groups were distributed in different immune states in the CGGA microarray cohorts and GSE16011 cohorts.
