# Supplementary material for: Multiomics Profiling and Clustering of Low-Grade Gliomas Based on the Integrated Stress Status
Source: Biomed Res Int. 2021 Jul 28;2021:5554436. doi: 10.1155/2021/5554436 (PMC8343268; doi:10.1155/2021/5554436)
Supplement: Supplementary 6 — Figure 2: the predictive accuracy of integrated stress risk model for LGG prognosis. [file 5554436.f6.pdf]

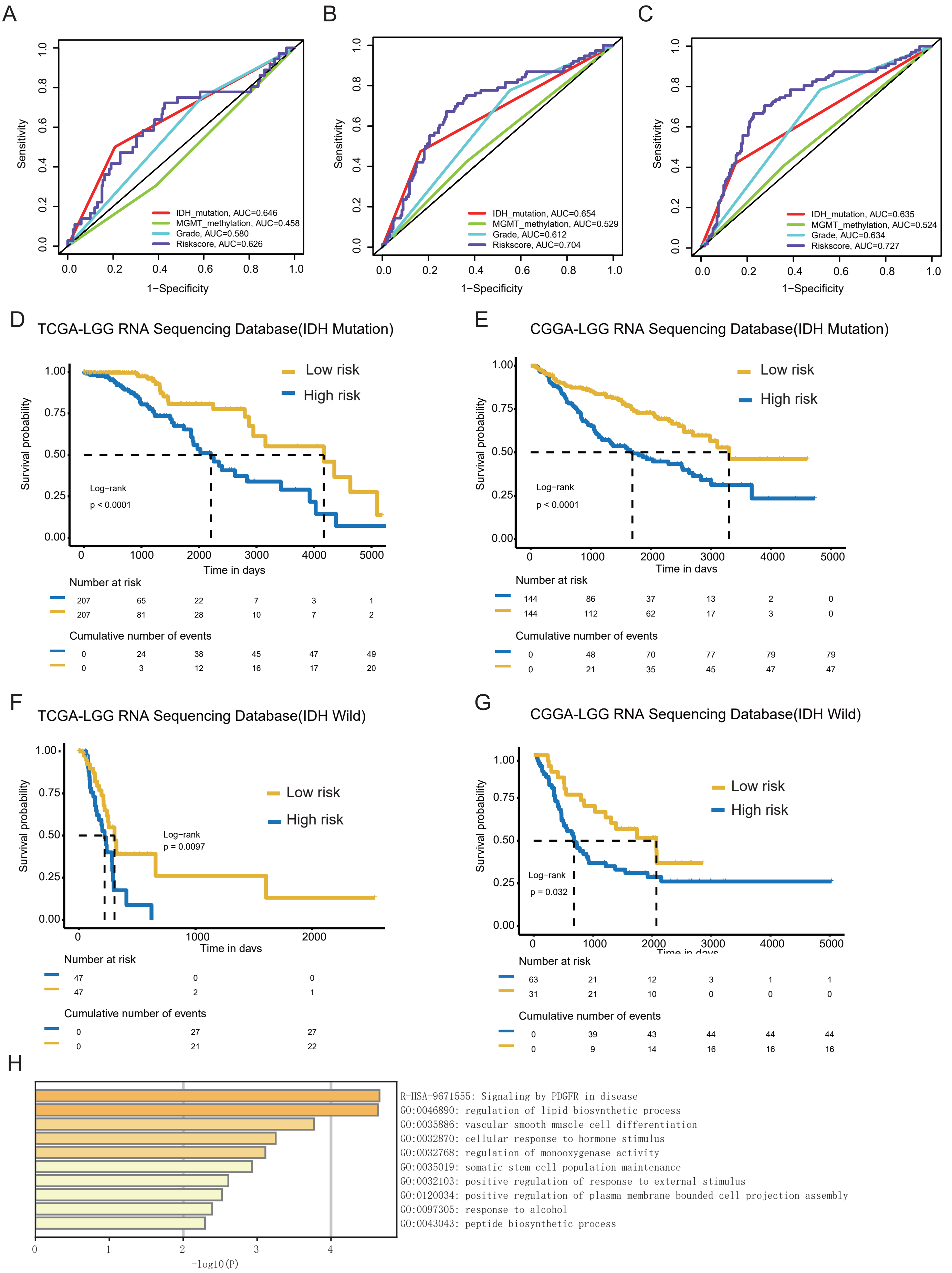

Supplementary Figure 2 The predictive accuracy of integrated stress risk model for LGG prognosis
(A-C) Risk score predicted 1 - year, 2 - year, and 3 - year of survival with ROC curve and AUC in TCGA. (D-G) High risk score showed predictive value in LGG cohorts stratified by IDH status in TCGA and CGGA.
(H) Enrichment analysis of corresponding gene sets of amplified genes.
