# Supplementary material for: Multiomics Profiling and Clustering of Low-Grade Gliomas Based on the Integrated Stress Status
Source: Biomed Res Int. 2021 Jul 28;2021:5554436. doi: 10.1155/2021/5554436 (PMC8343268; doi:10.1155/2021/5554436)
Supplement: Supplementary 7 — Figure 3: GO and KEGG enrichment (CGGA). [file 5554436.f7.pdf]

A

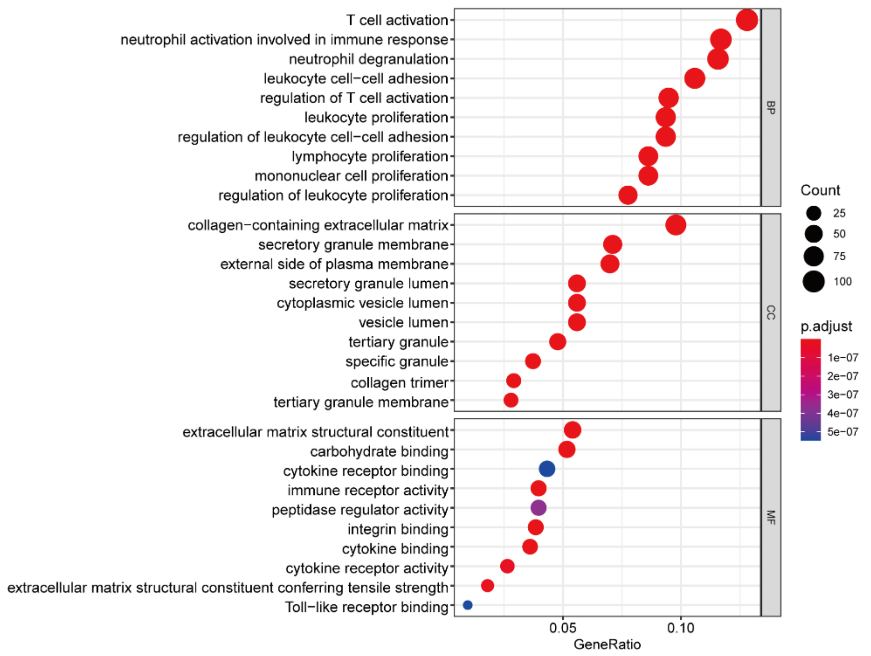

B

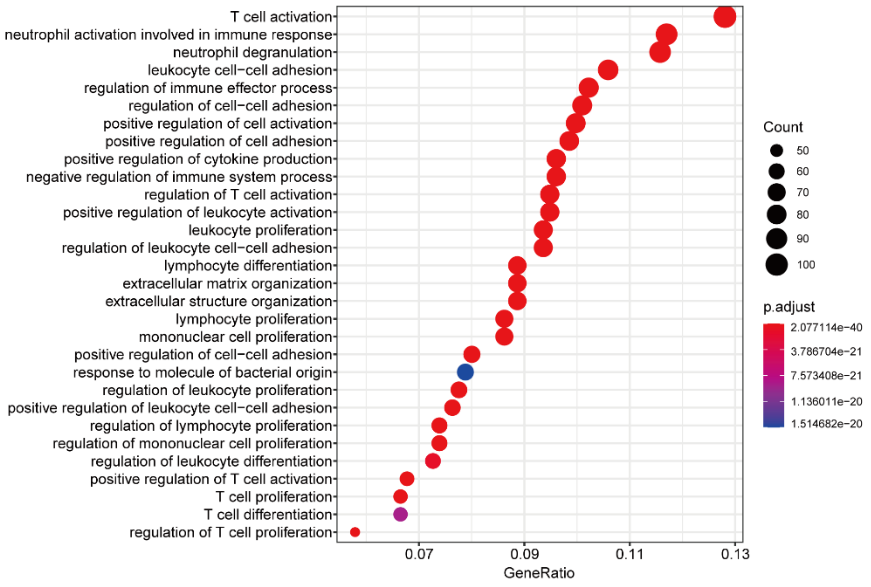

Supplementary Figure 3 GO and KEGG enrichment in CGGA(LGG).

(A) Results of GO enrichment of the differentially expressed genes between high risk group and low risk group in CGGA(LGG);(B) KEGG enrichment of the differentially expressed genes between high risk group and low risk group in CGGA(LGG).
